# Supplementary material for: HIVprotI: an integrated web based platform for prediction and design of HIV proteins inhibitors
Source: J Cheminform. 2018 Mar 9;10:12. doi: 10.1186/s13321-018-0266-y (PMC5845081; doi:10.1186/s13321-018-0266-y)
Supplement: Supplementary file 2 — Additional file 2. Source code of HIVProtI web server. [file 13321_2018_266_MOESM2_ESM.zip › HIVprotI_Source-code/hivprotI/weka-3-6-6/doc/weka/experiment/PairedStats.html]

PairedStats


---


|  |  |  |
| --- | --- | --- |
| **Overview** \| **Package** \| **Class** \| **Tree** \| **Deprecated** \| **Index** \| **Help** \| **Weka's home** | |  |
| **PREV CLASS**   **NEXT CLASS** | **FRAMES**    **NO FRAMES**     **All Classes** |  |
| SUMMARY: NESTED \| FIELD \| CONSTR \| METHOD | DETAIL: FIELD \| CONSTR \| METHOD |  |


---


## weka.experiment Class PairedStats

```
java.lang.Object
  weka.experiment.PairedStats
```

**All Implemented Interfaces:**: RevisionHandler

**Direct Known Subclasses:**: PairedStatsCorrected

---

``` public class PairedStats extends java.lang.Object implements RevisionHandler ```

A class for storing stats on a paired comparison (t-test and correlation)

**Version:**
:   $Revision: 1.10 $

**Author:**
:   Len Trigg (trigg@cs.waikato.ac.nz)

---

| **Field Summary** | |
| --- | --- |
| `double` | `correlation`             The correlation coefficient |
| `double` | `count`             The number of data points seen |
| `double` | `differencesProbability`             The probability of obtaining the observed differences |
| `int` | `differencesSignificance`             A significance indicator: 0 if the differences are not significant > 0 if x significantly greater than y < 0 if x significantly less than y |
| `Stats` | `differencesStats`             The stats associated with the paired differences |
| `double` | `sigLevel`             The significance level for comparisons |
| `Stats` | `xStats`             The stats associated with the data in column 1 |
| `double` | `xySum`             The sum of the products |
| `Stats` | `yStats`             The stats associated with the data in column 2 |


**Constructor Summary**

`PairedStats(double sig)`
  
          Creates a new PairedStats object with the supplied significance level.


| **Method Summary** | |
| --- | --- |
| `void` | `add(double[] value1, double[] value2)`             Adds an array of observed pair of values. |
| `void` | `add(double value1, double value2)`             Add an observed pair of values. |
| `void` | `calculateDerived()`             Calculates the derived statistics (significance etc). |
| `int` | `getDegreesOfFreedom()`             Gets the degrees of freedom. |
| `java.lang.String` | `getRevision()`             Returns the revision string. |
| `static void` | `main(java.lang.String[] args)`             Tests the paired stats object from the command line. |
| `void` | `setDegreesOfFreedom(int d)`             Sets the degrees of freedom (if calibration is required). |
| `void` | `subtract(double[] value1, double[] value2)`             Removes an array of observed pair of values. |
| `void` | `subtract(double value1, double value2)`             Removes an observed pair of values. |
| `java.lang.String` | `toString()`             Returns statistics on the paired comparison. |

**Methods inherited from class java.lang.Object**

`equals, getClass, hashCode, notify, notifyAll, wait, wait, wait`

**Field Detail**

### xStats

```
public Stats xStats
```

:   The stats associated with the data in column 1

---


### yStats

```
public Stats yStats
```

:   The stats associated with the data in column 2

---


### differencesStats

```
public Stats differencesStats
```

:   The stats associated with the paired differences

---


### differencesProbability

```
public double differencesProbability
```

:   The probability of obtaining the observed differences

---


### correlation

```
public double correlation
```

:   The correlation coefficient

---


### xySum

```
public double xySum
```

:   The sum of the products

---


### count

```
public double count
```

:   The number of data points seen

---


### differencesSignificance

```
public int differencesSignificance
```

:   A significance indicator:
    0 if the differences are not significant
    > 0 if x significantly greater than y
    < 0 if x significantly less than y

---


### sigLevel

```
public double sigLevel
```

:   The significance level for comparisons


**Constructor Detail**

### PairedStats

```
public PairedStats(double sig)
```

:   Creates a new PairedStats object with the supplied significance level.

    **Parameters:**: `sig` - the significance level for comparisons


**Method Detail**

### setDegreesOfFreedom

```
public void setDegreesOfFreedom(int d)
```

:   Sets the degrees of freedom (if calibration is required).

---


### getDegreesOfFreedom

```
public int getDegreesOfFreedom()
```

:   Gets the degrees of freedom.

---


### add

```
public void add(double value1,
                double value2)
```

:   Add an observed pair of values.

    :   **Parameters:**: `value1` - the value from column 1: `value2` - the value from column 2

---


### subtract

```
public void subtract(double value1,
                     double value2)
```

:   Removes an observed pair of values.

    :   **Parameters:**: `value1` - the value from column 1: `value2` - the value from column 2

---


### add

```
public void add(double[] value1,
                double[] value2)
```

:   Adds an array of observed pair of values.

    :   **Parameters:**: `value1` - the array containing values from column 1: `value2` - the array containing values from column 2

---


### subtract

```
public void subtract(double[] value1,
                     double[] value2)
```

:   Removes an array of observed pair of values.

    :   **Parameters:**: `value1` - the array containing values from column 1: `value2` - the array containing values from column 2

---


### calculateDerived

```
public void calculateDerived()
```

:   Calculates the derived statistics (significance etc).

---


### toString

```
public java.lang.String toString()
```

:   Returns statistics on the paired comparison.

    :   **Overrides:**: `toString` in class `java.lang.Object`
    :   **Returns:**: the t-test statistics as a string

---


### getRevision

```
public java.lang.String getRevision()
```

:   Returns the revision string.

    :   **Specified by:**: `getRevision` in interface `RevisionHandler`
    :   **Returns:**: the revision

---


### main

```
public static void main(java.lang.String[] args)
```

:   Tests the paired stats object from the command line.
    reads line from stdin, expecting two values per line.

    :   **Parameters:**: `args` - ignored.


---


|  |  |  |
| --- | --- | --- |
| **Overview** \| **Package** \| **Class** \| **Tree** \| **Deprecated** \| **Index** \| **Help** \| **Weka's home** | |  |
| **PREV CLASS**   **NEXT CLASS** | **FRAMES**    **NO FRAMES**     **All Classes** |  |
| SUMMARY: NESTED \| FIELD \| CONSTR \| METHOD | DETAIL: FIELD \| CONSTR \| METHOD |  |


---
